# Implementing National Institute for Health and Care Excellence smoke-free guidance in a secure facility: an evaluation of the prescribing costs in clozapine users

**DOI:** 10.1192/bjb.2017.16

**Published:** 2018-04

**Authors:** Stephen Noblett, Jane Beenstock, James Noblett, Joanne Ireland, Sarah Ormiston

**Affiliations:** 1 Lancashire Care National Health Service Foundation Trust, Preston, UK; 2 University of Sheffield, Sheffield, UK

## Abstract

**Aims and method:**

The increased rates of smoking in people with mental illness is well documented, and establishing smoke-free mental health environments has been emphasised over recent years. This article examines the financial costs of implementing smoke free guidance and assesses the cost associated with patients who were prescribed clozapine and who committed to stopping smoking cigarettes for the duration of the study period.

**Results:**

Patients (38) who were prescribed clozapine were included in the study. A moderate reduction in dose was noted with a moderate reduction in prescribing costs. The total increase in cost for the whole group, however, was £17 624, largely due to the use of nicotine replacement therapy and an increase in the number of clozapine assay tests.

**Clinical implications:**

Further studies on implementing this important policy change are needed. The positive effects must be balanced with increased financial pressure on Mental Health Trusts.

**Declarations of interest:**

None.

## Cost of smoking

Smoking imposes a huge economic burden on society. Action on Smoking and Health has concluded that the costs to society in England are approximately £13.9 billion per year. Around £2 billion of this is the cost to the National Health Service (NHS) of treating diseases caused by smoking.^1^ For adults with a common mental disorder such as depression and anxiety, smoking rates are almost twice as high compared to adults who are mentally well, and three times higher for those with schizophrenia or bipolar disorder.^2^ Smoking is thus a key contributor to the health inequalities that exist between people with a mental health disorder and the general population, which result in a difference in life expectancy of 15–20 years.^3^^,^^4^ Nationally there has been a growing recognition that providers of mental health services should be smoke free and support patients with nicotine addictions to stop smoking.^5^^–^^7^

## National Institute for Health and Care Excellence guidance

In particular, National Institute for Health and Care Excellence (NICE) guidance^5^ advocates that all NHS settings should be smoke free because of the health benefits to the individual, the wider community and healthcare providers. They emphasise that healthcare providers should promote healthy environments for their staff and patients. In this Trust the guidance was implemented through a nicotine management policy as, learning from other Trusts, the focus was on supporting people who have a nicotine addiction rather than banning smoking. The policy contents are similar to smoke-free policies in other Trusts: admitted smokers may no longer have smoking breaks and should manage their nicotine addiction through the use of nicotine replacement therapy (NRT) with behavioural support. Consequently, people admitted to smoke-free mental health units need to be supported to cease smoking tobacco cigarettes during their admission. Ideally, this would lead to permanent smoking cessation. Table 1 summarises some of the consequences in terms of cost or benefit to the individual and to the wider organisation.

**Table 1:** Summary of the costs and benefit consequences of smoking cessation following the introduction of a smoke-free policy in a mental health provider unit

Consequence	Cost or benefit?
Smoking cessation aids such as nicotine replacement therapy (NRT) and medications such as buproprion	Cost
Behavioural support^8^	Cost
Increase in number of clozapine assay tests required	Cost
Reduction in amount of antipsychotic medication required, with opportunity for fewer side effects from the medication	Benefit
Reduction in staff time spent supervising smoking breaks^9^	Benefit
Improvement in mental health^10^	Benefit
Reduction in premature mortality and in levels of morbidity	Benefit^11^^–^^13^
Potential lower costs of prescribed antibiotics for lung infections or other smoking-related physical health problems	Benefit
Public Health England estimates the long-term quit rate for long-term patients is 40%, and 25% for short-term patients	Benefit^14^
Public Health England estimates that the intervention could cost approximately £1430 per annum to the health and care system on average over 10 years	Cost^14^
Public Health England estimates net savings of approximately £1460 per annum per person to the health and care system on average over 10 years	Benefit^14^

## NRT and the associated costs and savings

NRT provides nicotine to the body without using cigarettes. The aromatic hydrocarbons found in cigarette smoke are known to increase the metabolism of some drugs due to induction of the cytochrome P450 enzyme system.^15^ NRT does not influence these metabolic enzymes.^16^ Plasma levels of clozapine are altered in the absence of tobacco smoke. Therapeutic-drug monitoring of clozapine is therefore useful with a recognised therapeutic range to regulate the required prescribing dose. Failure to monitor plasma levels in the context of stopping smoking may result in prescribing the incorrect dose. Following smoking cessation, clozapine doses may be reduced, decreasing the medication cost for the NHS in mental health. Smoking cessation could be financially beneficial to the NHS because the resources used to supervise smoking could be used elsewhere to improve health and well-being. This, however, may be offset by increased requirements for therapeutic-drug monitoring and the cost of NRT. Whereas there is extensive published literature demonstrating the long-term benefits of smoking cessation, in both the general population^17^ and in the population of people with a mental health disorder;^18^ there is limited published work describing the short-term costs and benefits.

## Aim

This project aimed to review the costs of providing NRT, clozapine and clozapine assay testing in the context of the new nicotine management policy within a secure mental health population. In January 2015, the secure mental health service implemented a nicotine management policy (NMP) in response to the publication of the NICE public health guidance 48.^5^ This evaluation focused on the costs and savings relating to clozapine and NRT because these are the two most direct expenditures from supporting people who smoke to manage their nicotine addiction without smoking tobacco cigarettes. NRT is required to manage the nicotine addiction and clozapine is commonly prescribed in the specialist services. Patients on clozapine who stop smoking are likely to have lower prescribing costs because of the need for lower dosages of medication when stopping smoking.^19^

## Method

### Setting

Guild Lodge is a secure mental health facility which provides care for 164 male and female patients within medium, low-secure and step-down ward environments. It also provides regional medium and low-secure care for males with acquired brain injury. The service provides a seamless transition of care between different levels of security in preparation for support in the community. The service is made up of 12 in-patient wards, 3 of which care for a total of 24 women. Ethical approval was not required as no patient information was identified. Advice was sought through the audit department within the Trust.

### Context

The study looked at a cohort of patients who were prescribed clozapine for a 12-month period at the time the NMP was implemented at Guild Lodge in January 2015. These people stopped smoking cigarettes at this time and maintained their smoke-free status for the duration of the study. This was monitored in the context of the secure in-patient and hospital grounds smoke-free environment. Prior to the implementation date, data was collected in an effort to try to anticipate where there may be particular risks due to elevated clozapine plasma levels for individual patients. Data included clozapine dose, plasma level (with current dose) and if any anticonvulsant medication was also prescribed.

The 6 month period prior to the implementation in January 2015 was considered in terms of dosage of prescribed clozapine and the number of clozapine assay tests undertaken. In the following 6 months, until July 2015, information relating to the dose of prescribed clozapine, number of clozapine assays and prescribed NRT was collated.

The total financial cost for this group, in the 6 months before and after implementation of the NMP, was calculated based on the cost to the organisation of clozapine, clozapine assay tests and specific NRT, including patches, lozenges and inhalators.

## Results

A total of 38 patients were included in the study, representing 23% of the total in-patient population. Of these, 8 were women (21%) and 30 were men (79%). Data was obtained from each of the 12 wards within the service. In total, 48 out of 164 patients were prescribed clozapine in the service at the time. Ten of these were non-smokers and were therefore not included in the study. At the time of the study, the hospital site became a smoke-free environment, although there was occasional evidence of illicit smoking within the secure setting.

The results demonstrated a small reduction of 6.5% in mean clozapine dose from 381 mg to 356 mg in the 6 month period following the NMP implementation. However, the number of clozapine assays carried out in this period increased by 200%, with the mean number of assays rising from one to three. The increased number of clozapine assays had a significant financial effect (Table 2), although the number of required assays is likely to reduce as plasma levels stabilise following smoking cessation.

**Table 2:** Total cost before and after implementation of National Institute for Health and Care Excellence smoke-free guidance

	Total cost of clozapine dose before intervention (£)	Total cost of clozapine dose after intervention (£)	Total cost of assays before intervention (£)	Total cost of assays after intervention (£)	Total cost of nicotine replacement therapy (£)	Total cost before intervention (£)	Total cost after intervention (£)	Total difference in cost after 6 months (£)
Total cost for service	7489.41	6991.44	878.75	2280	16 818.96	**8368.16**	**26** **090.40**	**17** **623.66**
Mean cost per patient	197.09	183.99	23.13	60	442.60	**220.21**	**686.59**	**463.78**
Range of individual costs	77.61–362.18	77.61–362.18	0–71.25	23.75–142.50	0–871.92	**101.36–385.93**	**127.23–1184.25**	**0–930.24**

Table 2 demonstrates the specific prescribing costs for medication and for clozapine assays for the service, as advised by the pharmacy department. These figures are based on the costs of clozapine over the two 6 month periods for the organisation, costs for each assay test and total cost of NRT. Costs did not include any additional phlebotomy or laboratory costs.

A total of 5 out of the 38 patients had no clozapine assay tests taken in the 6 month period prior to stopping smoking, which may have represented clinical stability and consistent dose prescribing in this group. A total of 28 patients (74%) were prescribed NRT throughout the 6 month period with the majority using patches (53%) and inhalators (45%). This resulted in a cost of £16 819 for the 6 month period.

There was only a moderate reduction in the cost of prescribed clozapine because of limited dosage change, but also due to the relative in-expense of clozapine itself. For 18 out of the 38 patients, the cost of clozapine remained the same, indicating that the dose was unchanged during this period. Although the patients had committed to stopping smoking, it is possible that they continued to smoke cigarettes at times, leading to an increased cost of prescribed NRT, as this may have had little clinical benefit for these individuals.

For 35 patients, representing 92% of the sample, there was an increased cost in the 6 month period following the implementation of the NMP, with a total cost of £18 641.66 for the whole group during this time.

## Discussion

This study showed that in the 6 months following the introduction of the NMP in this unit there was a slight decrease in the costs from prescribed clozapine, and increased costs generated by more assay tests and the use of NRT.

These results will have been influenced by the take-up rate of NRT patients, and the extent to which they were compliant with not smoking cigarettes during the 6 month period. This will have affected the amount of NRT prescribed and the consequent impact on clozapine plasma levels. Current and future costs will also be affected by the number of patients who use e-cigarettes.

The current culture and practice around smoking has been evolving since the 6 months reviewed in this study. Recently, the use of e-cigarettes has been piloted and a much more robust approach to implementing the NMP has affected attitudes and behaviours in relation to the NMP. In addition, patients have been given less time in the grounds where they have been likely to smoke cigarettes, especially when they have access to the community on leave. This may begin to change the culture of leave within the grounds to be more therapeutic and less about gaining access to cigarettes.

This study has not included all the financial costs that could be affected by the introduction of the NMP, such as prescription costs for physical health problems related to smoking, and staffing costs for supervising patients who have smoking breaks.

Although there is good evidence that the overall benefits of smoking cessation are greater than costs for both individuals and society in the longer term, in the short term there are some immediate financial pressures generated for Mental Health Trusts. Further work is needed to understand if these results are likely to be the same for other Trusts implementing this important policy change that is needed to improve the mental and physical health of people using mental health services. Smoke-free policies challenge the culture in mental health units,^20^^–^^23^ but the financial pressure involved should not derail the ambition to be smoke free because it is consistent with national policy and is of significant benefit to people with a mental health disorder.^18^

## About the authors

**Stephen Noblett** is a consultant forensic psychiatrist and **Jane Beenstock** is a consultant in public health at the Lancashire Care National Health Service (NHS) Foundation Trust in Preston, UK. **James Noblett** is a bachelor of arts student of economics at the University of Sheffield, Sheffield, UK. **Joanne Ireland** is a Deputy Lead Pharmacist and **Sarah Ormiston** is a Clinical Audit Facilitator, both in Specialist Services at the Lancashire Care NHS Foundation Trust in Preston, UK.
